# The interior climate and its microclimatic variation of temperate forests in Northern Patagonia, Argentina

**DOI:** 10.1007/s00484-024-02617-5

**Published:** 2024-01-27

**Authors:** Alois Simon, Jonas Fierke, Ernesto J. Reiter, Gabriel A. Loguercio, Steffi Heinrichs, Birgitta Putzenlechner, Natalia Z. Joelson, Helge Walentowski

**Affiliations:** 1 Faculty of Resource Management, HAWK University of Applied Sciences and Arts, Göttingen, Germany; 2 Department of Cartography, GIS and Remote Sensing, Institute of Geography, University of Göttingen, Göttingen, Germany; 3 Plant Ecology and Ecosystems Research, University of Göttingen, Göttingen, Germany; 4 Andean Patagonian Forest Research and Extension Center (CIEFAP), Esquel, Argentina; 5 Faculty of Engineering, Department of Forestry, National University of Patagonia San Juan Bosco, Comodoro Rivadavia, Argentina; 6 Silviculture and Forest Ecology of the Temperate Zones, University of Göttingen, Göttingen, Germany; 7 Faculty of Biology and Psychology, University of Göttingen, Göttingen, Germany

**Keywords:** Air temperature, Stand microclimate, Vapour pressure deficit, Elevational zonation, Successional stages, Mountain forests

## Abstract

Knowledge on mesoclimatic zonation and microclimatic variations within mountain forest ecosystems is crucial for understanding regional species turnover and effects of climate change on these systems. The temperate mountain forests in the Andean region of South America are among the largest and contiguous natural deciduous forest areas in the world. Due to their pronounced disturbance regime and different successional stages, a climatic zonation combined with the characterisation of its microclimatic variation is important to identify thresholds of species occurrences.

We used micro-loggers to measure air temperature and relative humidity for one year at 40 measurement locations along longitudinal and elevation gradients in mountain forests in Northern Patagonia, Argentina. Our results unveil mesoclimatic patterns within these forests characterised by variations in temperature and vapour pressure deficit along the elevational gradient in general, but also at different times of the year. For example, *Austrocedrus chilensis* and *Nothofagus dombeyi* forests differed mainly by temperature and its diurnal range in the warmest months of the year. Also, differences between forest stands and gaps were more pronounced in the warmest months of the year and at lower elevations, with up to 2.5 K higher temperatures in the second half of the day in gaps. We found clear indications that shrubland of *Nothofagus antarctica* representing a successional stage after disturbances alters the mesoclimatic pattern, favouring forest fire ignition. Such mesoclimatic variations have a major influence on tree species turnover and ecological processes within these forest ecosystems.

The findings contribute to our understanding of the complex interplay between topography, climate, and vegetation in shaping the spatial patterns of species occurrences.

**Supplementary Information:**

The online version contains supplementary material available at 10.1007/s00484-024-02617-5.

## Introduction

The landscape of Northern Patagonia provides a paradigm for studying the relationship between climate, climate-driven disturbances and vegetation development. According to Pollmann (2001), climate and microclimatic factors (e.g. temperature and humidity) are the most important ecological factors determining the floristic differentiation of Patagonian Andean *Nothofagus* forest communities. There are two main reasons for this strong climatic impact. First, a distinct longitudinal zonation extends from the humid Valdivian rainforests of the western Andes, through various progressively drier forests, woodlands and shrublands, to the semi-arid steppe of Northern Patagonia, within just 80–100 km. This west-east differentiation is most pronounced at lower to mid elevations (Amigo and Rodríguez Guitián 2011) and represents one of the steepest plant productivity gradients on earth (Kitzberger et al. (2022). This climatic gradient is associated with a shift in the annual temperature range (thermal continentality) (Daniels and Veblen 2003), which strongly shapes the distribution of tree species (Fang and Lechowicz 2006; Amigo and Rodríguez Guitián 2011). Second, there is a significant elevational zonation caused by a decrease in temperature and an increase in precipitation, mountain fog and in the amount of snow. Moreover, the west-east moisture gradient is amplified by the differences between mesoclimatic conditions of south- and north-facing slopes (Donoso et al. 2014) and effects of topography (e.g. concave or convex topography). In combination, this can lead to more humid or drier site conditions, favouring tree growth and selection pressure depending on the species (Stage and Salas 2007; Diaz et al. 2020; Oddi et al. 2022).

The main climatic gradients result in contrasting vegetation types and associated disturbance regimes, which in turn lead to pronounced local variations in the forest microclimate and alterations of the mesoclimatic pattern. The western sectors are dominated by evergreen *Nothofagus dombeyi* ((Mirb.) Oerst.) and harbour species adapted to moist oceanic conditions. These forest types experience small-scale treefall gaps in low to mid elevations, snow breakage in higher elevations, and infrequent yet intense forest fires (Sagarzazu and Defossé 2009). On the other hand, frequent low-intensity forest fires and drought-induced mortality are the predominant disturbance agents in drier regions that are located in the east (Kitzberger 2012). Eastern areas are dominated by xeric coniferous forest of *Austrocedrus chilensis* ((D. Don) Pic.-Ser. & Bizz), a species with high warmth requirements and rarely found above 900 m a.s.l. in the southern part of its natural range. Following disturbances in the eastern area, *Nothofagus antarctica* ((Forst.) Oerst.) emerges as a successful species forming dense clonal shrublands. While the increasing drought stress towards the east has a selective effect in the lower to middle elevations, it is frost and snow during winter in higher elevations. The subalpine belt, characterised by a temperate oceanic mountain climate with periodic snow, is thus dominated by *Nothofagus pumilio* ((Poepp. & Endl.) Krasser) across the trans-Andean catena from west to east. It is bare in winter, has a high stem elasticity, and can grow both high-stemmed and as a krummholz (Hildebrand-Vogel et al. 1990). The species is thus particularly well adapted to the mechanical stresses of wet snow, especially in La Niňa periods in which more snowfall is registered (Cortés and Margulis 2017).

Given that even small-scale variations in the terrain climate situation can lead to altered adaptation pressures and thus altered species composition, the importance of reliable information on meso- and microclimate becomes evident (Bramer et al. 2018; De Frenne et al. 2021). In particular, tree regeneration, understorey plants and soil-atmosphere processes operate within climatic conditions that significantly diverge from those of free-air measurements. The importance of incorporating microclimate into species distribution models is also widely recognised, and Lembrechts et al. (2019) provide a summary of applications. Accurate site data, including the interior forest climate (i.e. microclimate), are essential to understand the current distribution of the abovementioned species particularly in the context of disturbances. However, the complex three-dimensional meso- and microclimatic variability in space and time is contrasted by the small number of weather stations, as it is characteristic in mountain regions (Thornton et al. 2022). While high-resolution information on interior temperatures is available for Europe’s forests (Haesen et al. 2021; Haesen et al. 2023), such studies are rare in our study region. The network of weather stations (free-air measurements) in the montane forest belt of the eastern slopes of the Andes is fragmented and lacks reliable long-term measurements (Condom et al. 2020; SMN 2023; SNIH 2023). Similar to the problem pointed out by Young and León (1999) for the Peruvian Andes, all information on data of biologically significant climate variables, as proposed, e.g. by the WorldClim dataset (Fick and Hijmans 2017), is usually derived from extrapolations from stations at lower or higher elevations, other situations (deviating topography) or from other sections of the Andes. Beside mean values for climatic periods, there are also hardly any measurement campaigns on the meteorological annual course of the weather. This lack of data is presumably caused by the difficult accessibility of remote mountain forest belts.

With this study, we aim to contribute to the understanding of climate gradients across the temperate mountain forest in the Andes of Northern Patagonia and provide information for species distribution models in the context of climate change and disturbance regimes. Therefore, our hypotheses are that (i) the gradient of the annual range of temperature (thermal continentality) increases linearly along the longitudinal extent of the study area from west to east, (ii) the vegetation types investigated show distinct thermal niches along the elevation transects, and (iii) disturbance-induced mesoclimatic variations allow conclusions to be drawn about pre-disturbance vegetation types and regeneration pathways.

## Methodology

### Study site and measurements

**Fig. 1:**
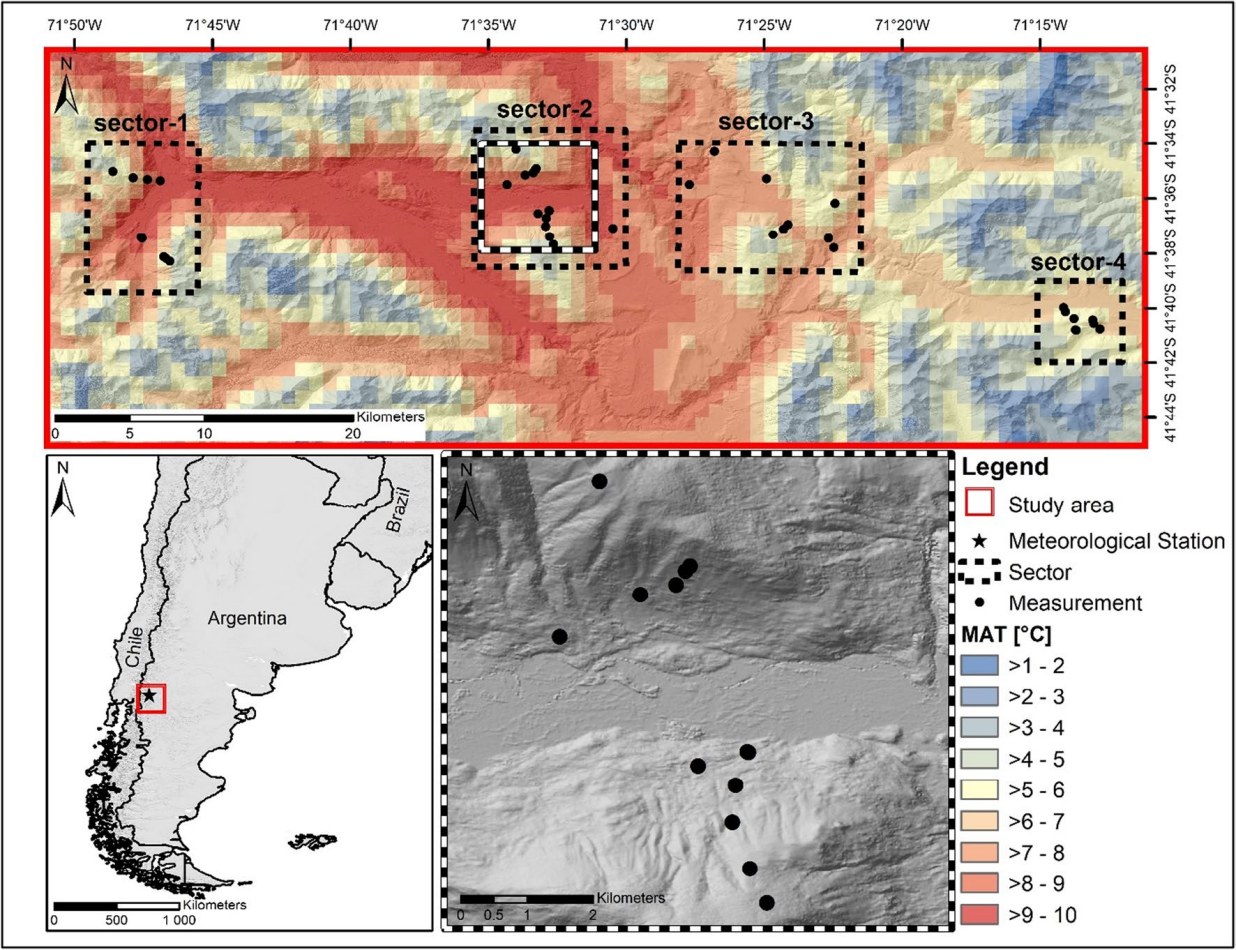
Location of the study area, different sectors and an elevational transects (sector-2). MAT, mean annual temperature from WorldClim 2.1 (Fick and Hijmans 2017)

The study area extends from the Valle El Manso in the west to the eastern edges of the mountains close to Río Chico at a latitude of 41°34′ to 41°41′ South and a longitude from 71°50′ to 71°10′ West (Fig. 1) and is located in the Province of Río Negro. To cover the strong mean annual precipitation gradient from west (sector-1, appr. 1960 mm) to east (sector-4, appr. 760 mm) of about 1200 mm (Karger et al. 2017), the study area is separated into four sectors. As the main valley is west-east orientated, the elevational transects were located at north and south facing slopes at each sector. The decisive site factors, connected with aspect, are strongly varying solar radiation input (Oddi et al. 2022) and the exposure to prevailing Westerlies (Kalela 1941). The eight elevational transects ranged from 500 to 1600 m a.s.l. and covered four main vegetation types. These vegetation types are characterised by the following tree species: (i) *Austrocedrus chilensis* dominated dry forests in lower elevations (6 measurement locations, 600–890 m a.s.l.), (ii) *Nothofagus dombeyi* dominated mesic pure and mixed forests in low to mid elevation (7 measurement locations, 500–1000 m a.s.l.), (iii) *Nothofagus antarctica* successional stages of post-fire shrublands in mid-elevation slopes (9 measurement locations, 900–1400 m a.s.l.), and (iv) *Nothofagus pumilio *dominated subalpine deciduous forests (15 measurement locations, 1100–1600 m a.s.l.).

For selecting the measurement locations along the transects (Fig. 1), we started at the valley bottoms at the first 100 m contour line with the respective vegetation type. Each time the vegetation type changed along the transect, a measurement location was selected at the next 100 m contour line. The transects ended at the tree line and were completed with a measurement location at the uppermost 100 m elevation step within the forest. Following this methodology, the measurement locations describe the lower and upper edges of the species distributions along the transects. In addition to measurements in the forest stands (interior climate), measurement locations at western edges (protected from prevailing wind direction) of forest gaps were selected, too. Each of the gaps measured at least one diameter of the height of the surrounding forest stand and was selected next to a related forest stand measurement.

The measurements were conducted between February 2022 and March 2023, and the recordings took place 6 times a day at 02:00, 06:00, 10:00, 14:00, 18:00, and 22:00 o’clock Argentina Time (ART) which corresponds to UTC-3. We used iButton DS1923 Hygrochron micro-loggers (Analog Devices, Inc., USA) with a sensor accuracy for temperature of at least ± 0.5 °C with a resolution of 0.0625 K (16 bit) and for relative humidity of ± 5% with a resolution of 0.6% (8 bit) (Analog Devices 2023). The sensors were mounted on the east side of trees at 2 m height and protected against direct radiation and weather conditions (e.g. rain, snow, prevailing West winds) with an upturned white plastic cup of 250 cm^3^ volume placed over the sensor. The sensor was placed appr. 2 cm outside the cup and had at least 1 cm distance to the bark, to allow for sufficient air turbulence. In doing so, we followed the methodology described by Hohnwald et al. (2020). In total, we placed 44 micro-loggers along the 8 elevational transects.

### Data analysis

We selected a one-year period (365 days) between 1 March 2022 and 28 February 2023 from the measurements for the analysis. In order to categorise the recording period of our micro-loggers in relation to the climate normal from 1991 to 2020, we used the records from the weather station in San Carlos de Bariloche (SMN 2023) at 842 m a.s.l., which is approximately 60 km away from the study site (Fig. 1). Due to the differences between free-air measurements from meteorological stations and sub-canopy measurements (Haesen et al. 2021), and the large spatial distance (San Carlos de Bariloche) or coarse spatial resolution (ERA5-Land) (Muñoz-Sabater et al. 2021) of available data, no direct comparison of the values was conducted.

From the recorded four-hourly data, we calculated different climatic variables in line with global available bioclimatic variables (Fick and Hijmans 2017; Karger et al. 2017). We defined thermal continentality as the difference of the Mean Monthly Temperature (MMT) between the warmest and the coldest month per site according the bioclimatic variable BIO7 (Fick and Hijmans 2017). Since the thermal continentality is subject to an elevational trend (Rivas-Martínez et al. 2011), we fitted a linear regression model to the original values and used the differences to an elevation of 1000 m a.s.l. to standardise the continentality to that elevation. For describing the diurnal range of temperature (DRT), the daily minimum and maximum values are used (bioclimatic variable BIO2). The stand-gap differences are the deviation from the stand values (interior climate). We calculated the vapour pressure deficit (VPD) from the recorded air temperature and the relative humidity. VPD is a direct measure of the atmospheric demand for water and was found to be a reliable indicator for dead fuel moisture content in different forest ecosystems (Resco de Dios et al. 2015) We used the following expression:

Vapour Pressure Deficit (VPD) [hPa] = vp_sat_ × (1 − relative humidity [%]/100)

where vp_sat_ is the saturation vapour pressure calculated with the equation proposed by Murray (1967), provided by the R package: ‘humidity’ (Jun 2019). To characterise the non-linear elevation trend of daily mean VPD, we used a simple generalized additive model (GAM) (Wood 2017). To describe the alteration of the VPD by *N. antarctica–*dominated successional stages, we fitted a model to the VPD of the other vegetation types and compared it to the values of *N. antarctica*. Furthermore, we calculated a forest fire ignition threshold according to Paritsis et al. (2015) derived from recorded forest fires of Sagarzazu and Defossé (2009) for comparison with the pattern of the VPD. It is defined as days with maximum daily temperature >25 °C and minimum relative humidity <25%.

For analysing the general characteristics of the measurements, we applied descriptive and multivariate statistics. All data analysis was done with the R statistical software (R Core Team 2019). We calculated daily, monthly means and the annual mean with the R package: ‘xts’ (Jeffrey A. Ryan and Ulrich 2018) from the six recordings per day. We tested the data for normal distribution with the Shapiro-Wilk test and interpretation of normal qq-plots. For normal distributed data, we used the *t*-test from the R package: ‘stats’ (R Core Team 2019). For non-normal distributed data, the Kruskal-Wallis tests with post hoc Nemenyi tests (method = Tukey) from the R package: ‘PMCMR’ were used (Pohlert 2014). For graphical display, the R packages: ‘lattice’ (Sarkar 2008), ‘ggfortify’ (Tang et al. 2016), ‘oce’ (Kelley and Richards 2022), and ‘ggplot2’ (Wickham 2016) were used.

## Results

### Categorisation of the recording period

In the meteorological station of San Carlos de Bariloche, mean monthly temperatures between March and August 2022 (in our recording period) were consistently below the reference period (1991–2020) with highest deviations in March (−1.19 K). Conversely, temperatures from September 2022 to February 2023 exceeded the reference period, with the highest anomaly in November (+3.79 K), surpassing the confidence intervals of ± 2 sigma (supplement Figure SF1). The annual difference between the reference and the recording period amounts 0.28 K, classifying the recording period as a positive anomaly, with particular emphasis on the months from September 2022 to February 2023 (SMN 2023).

The climate dynamics of northern Patagonia are influenced by at least two significant climate phenomena—the Southern Annular Mode (SAM) and the El Niño Southern Oscillation (ENSO). With respect to the SAM, our analysis identified 277 positive and 88 negative days based on the Antarctic oscillation index during the recording period. A positive SAM phase is associated with anomalously warm and dry conditions over southern South America (Silvestri and Vera 2003; Gillett et al. 2006) exerting substantial impacts on the disturbance regimes and ecosystems of Patagonia (Holz and Veblen 2011; Holz et al. 2017). Turning to the ENSO, all months of the recording period were characterised by a positive Southern Oscillation Index (SOI), indicative of a La Niña event (Trenberth 1984; NOAA 2023). The influence of ENSO is regulated by numerous factors, complicating the prediction of its seasonal effects (Cai et al. 2020). However, late stages of La Niña events and an increased ENSO activity are associated with prolonged droughts and hot summers, affecting water availability and fire activity within the region (Kitzberger and Veblen 1997; Villalba and Veblen 1998).

### Elevational zonation

From the 44 selected measurement locations, we obtained 40 complete time series, i.e. 9% of the micro-loggers have not recorded completely. In total, we have recordings in forest stands dominated by *A. chilensis* (6), *N. dombeyi* (7), and *N. pumilio* (15), successional stages of post-fire shrublands of *N. antarctica* (9), and recordings in gap locations associated to forest stands (3). Due to disturbance-induced changes in stand structure during the measurement period, we were only able to evaluate stand-gap comparisons for *A. chilensis* and *N. pumilio*.

We found an elevation-dependent mean annual temperature (MAT) lapse rate of −0.47 K 100 m^−1^ (Table ST1) across all forest stand measurements and sectors. The individual analysis of the elevation dependency of the four sectors (west to east) and the two aspects (north-northwest and south-southeast) shows only a slight increase from west to east and minor differences between the lapse rates of the two aspects. However, the intercepts of the individual linear regression models indicate higher temperature values at the northern aspect, with high radiation input. The variance explained by the individual models is very high with an adj. *R*^2^ of 0.87 (north-northwest) and 0.97 (south-southeast) with *p* < 0.0001, respectively. Based on the identified lapse rate of all our measurements, this corresponds to approximately to 150 m, 115 m and 65 m elevation differences between the two aspects at 500 m a.s.l., 1000 m a.s.l. and 1500 m a.s.l., respectively.

**Fig. 2:**
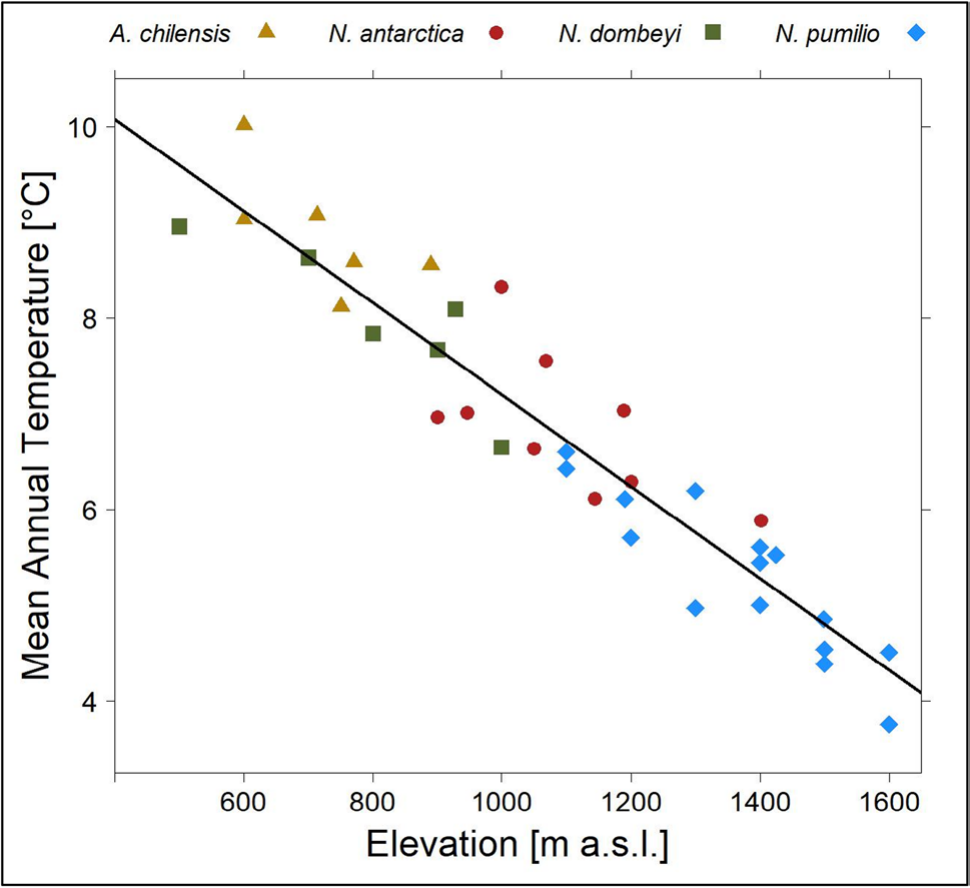
Vegetation type distributions along elevational gradients (without gaps)

From Fig. 2, we observed a clear differentiation between *N. dombeyi* (small evergreen, mesomorph leaves) and *N. pumilio* (deciduous), the niche of post-fire shrublands of *N. antarctica* (deciduous) between these two species and the dominance of *A. chilensis* (evergreen scale-like, xeromorph leaves) and *N. dombeyi* in the lower elevations with higher temperatures. These differentiations were confirmed by the significant differences in the mean daily temperature (MDT) given in Table 1. To identify the individual thermal characteristics of the two species at the lower elevations, we analysed the MDTs and DRTs in the warmest (WQ) and coldest (CQ) quarter of the year and found significant differences between them, in terms of the MDT and DRT in the warmest quarter (Table 1 and supplement Figure SF2).

**Table 1:** Summary and statistical comparison of the mean daily temperature (MDT) and diurnal range of temperature (DRT) of different vegetation types. Mean ± standard deviation; dif letters indicate significant differences based on adjusted *p*-value <0.001 (Kruskal-Wallis test with post hoc Tukey test)

Vegetation types	365 days		Warmest quarter (WQ, Dec.–Feb.)		Coldest quarter (CQ, Jun.–Aug.)		Warmest quarter (WQ, Dec.–Feb.)	
	MDT	dif	MDT	dif	MDT	dif	DRT	dif
*A. chilensis*	8.9 ± 6.3	a,b	16.0 ± 3.6	a	2.06 ± 2.0	a,b	11.2 ± 4.5	a
*N. dombeyi*	8.1 ± 6.1	b,a	14.8 ± 3.6	b	1.69 ± 2.0	b,a	9.7 ± 4.3	b,d
*N. antarctica*	6.9 ± 6.1	c	13.2 ± 4.0	c	0.71 ± 2.6	c	12.8 ± 5.8	c
*N. pumilio*	5.3 ± 6.0	d	11.3 ± 4.3	d	−0.51 ± 2.7	d	8.9 ± 3.7	d,b

### Longitudinal zonation

**Fig. 3:**
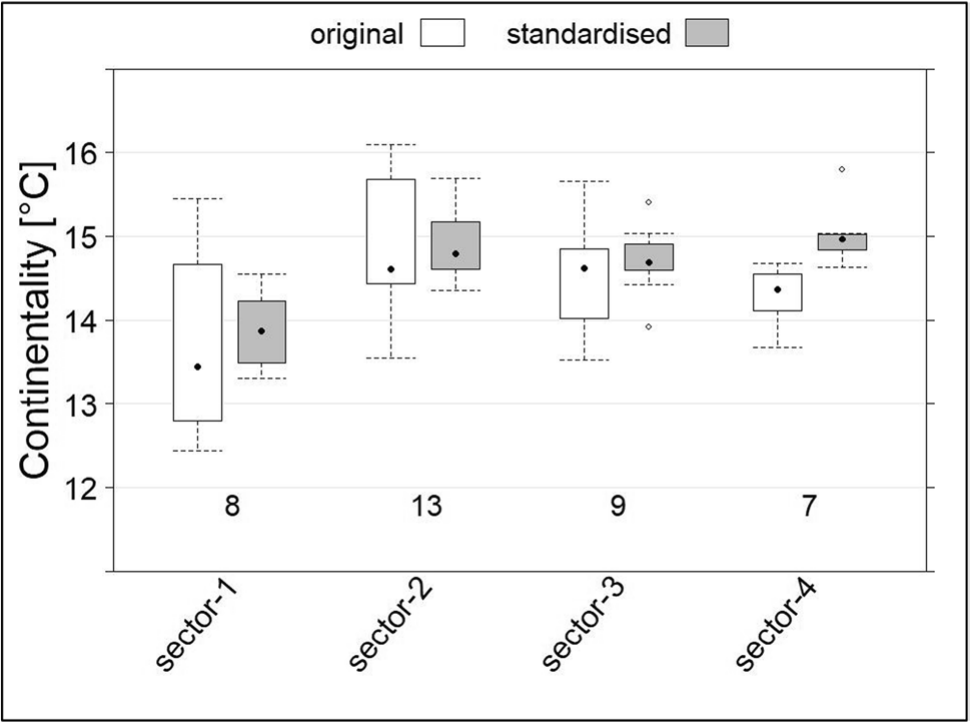
Change of thermal continentality in the study area from west (sector-1) to east (sector-4). Original, measurements at elevation; standardised, values standardised to 1000 m a.s.l.; points, median; whisker, maximum of 1.5 interquartile range; numbers indicate count of recordings

In addition to the elevational gradient, we identified a strong continentality gradient in the study area from west to east (Fig. 1). Thereby, the differences between the sectors are masked by an elevational trend (cont [°C] = −0.0022 × elevation [m asl] + 16.9052; adj. *R*^2^ = 0.59, *F*-statistic (df = 35) 54.4, *p* < 0.0001). The standardised continentality allowed us to compare the different sectors that are characterised by different elevations of valley bottoms (Fig. 3).

**Fig. 4:**
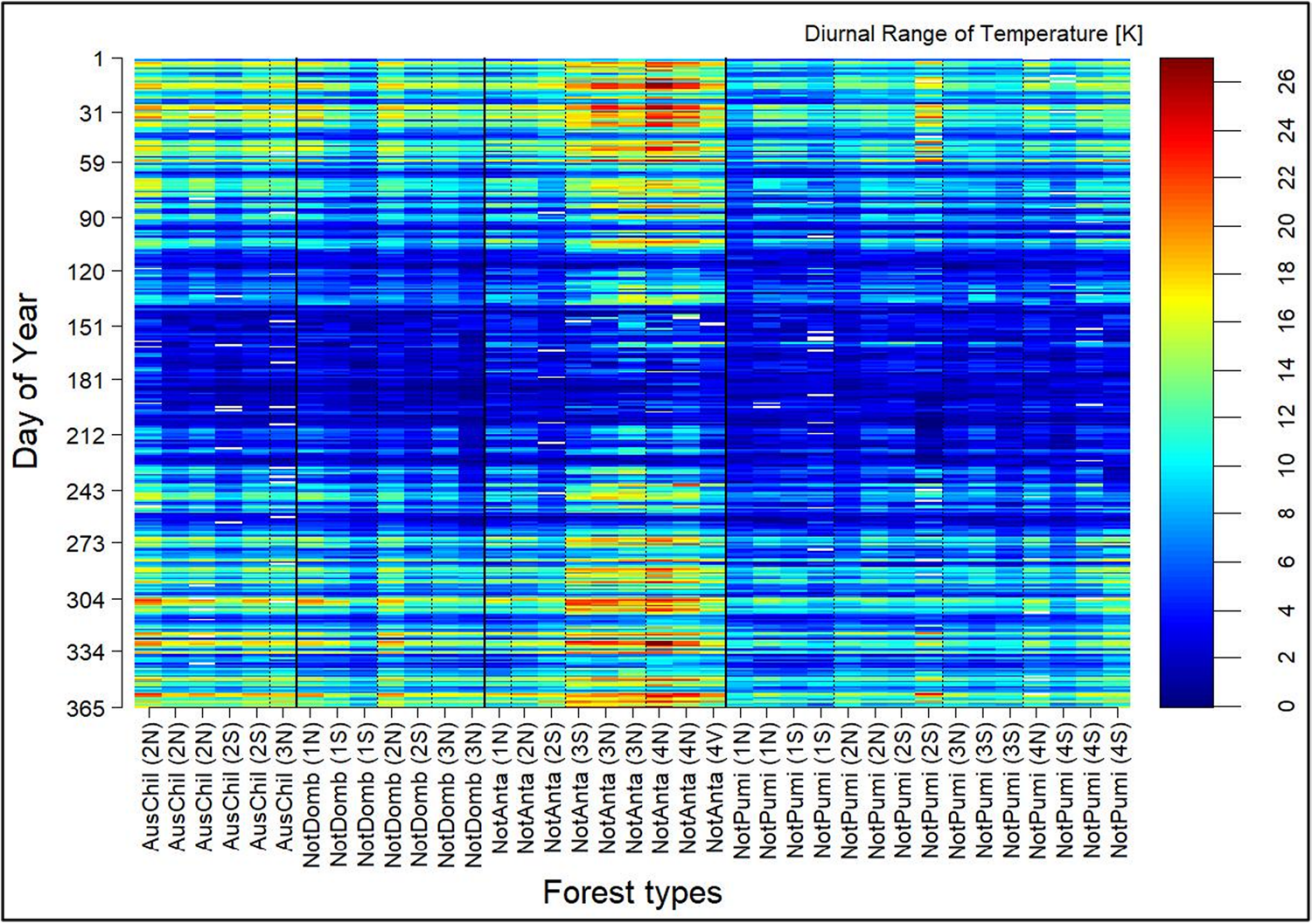
Temporal pattern of the diurnal range of temperature (DRT) of the different vegetation types (without gaps). X-axis: AusChil, *A. chilensis;* NotAnta, *N. antarctica*; NotDomb, *N. dombeyi;* NotPumi, *N. pumilio*; in brackets: sector (1–4) and aspect (N, north; S, south; V, valley bottom); sorted by sector (dashed line), aspect and elevation; see also Fig. 1

In comparison to the original measurements, the standardised thermal continentality showed the similarity between sector 2, 3, and 4. Only sector 1, which is the most western one (Fig. 1), showed a clearly lower thermal continentality. The clear difference between sector 1 and the others is also visible in the temporal pattern of the DRT (Fig. 4), e.g. for *N. pumilio* and *N. antarctica* which are present in all four sectors. Fig. 4 shows the temporal pattern in the one-year measurement period for each sample location, which each horizontal bar indicating the mean DRT value of the day of the year. Thereby, the DRT increased from west to east and in general with elevation. Likewise, the VPD increases from west to east (supplement Figure SF3) and manifests the described continentality gradient.

### Micro- and mesoclimatic variation within the vegetation types

**Fig. 5:**
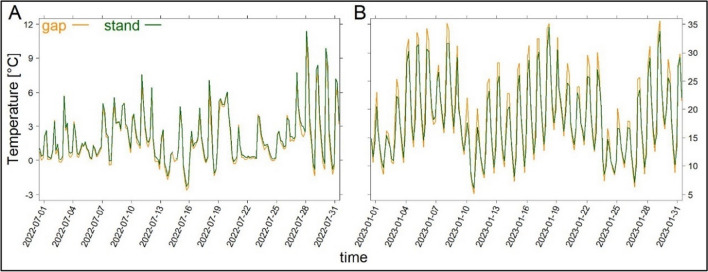
Comparison of the diurnal variation of temperature between a forest stand and a gap measurement location for *A. chilensis* for the coldest month (**A**) and warmest month (**B**) (see Table ST2)

We found significant differences between gaps and stands in *A. chilensis* at the warmest month (Fig. 5B) and at measurement times 10:00 and 14:00 o’clock, only. The results of the two-sided *t*-tests are given in supplement Table ST2. During this time of the day, the temperatures were up to 2.5 K higher in the gap. For *N. pumilio,* higher temperatures occurred in the gap in the afternoon (14:00, 18:00), however, being not significant. For the rest of the day, especially during night, the temperatures were higher in the stand for both species, leading to a more balanced stand microclimate compared to the gap. This is also confirmed by the stand-gap differences in DRT (supplement Figure SF4, Table ST3). As with temperature, the differences in DRT were only present during the warmest months and the deviations between stands and gaps were greater for *A. chilensis* than for *N. pumilio*.

**Fig. 6:**
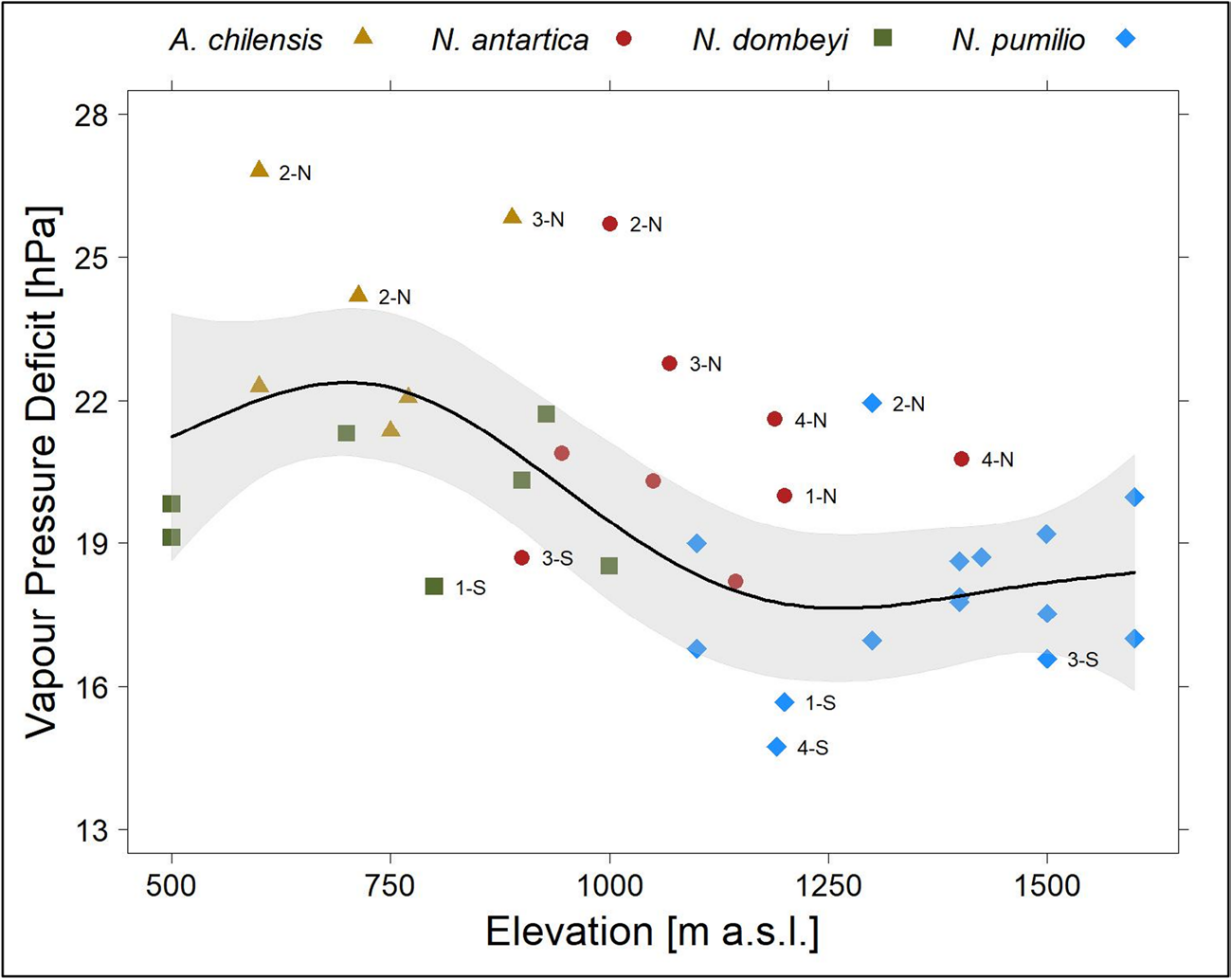
Annual maximum of the daily mean vapour pressure deficit (VPD) for the different vegetation types. Line, fitted model; shaded area, 95% confidence intervals (1.96 times standard error); labels, sector (1–4) and aspect (N, north; S, south)

Besides the temperature variation due to small-scale disturbance gaps, we paid particular attention to the differences in VPD caused by the different vegetation types and the influence of successional stages following large-scale disturbances. The annual maximum of the daily mean VPD values (Fig. 6) was highest for *A. chilensis* (23.8 ± 2.2), followed by *N. antarctica* (21.0 ± 2.2) and *N. dombeyi* (19.8 ± 1.4) and lowest for *N. pumilio* (17.9 ± 1.8) forest stands (hPa, mean ± standard deviation). The elevational trend of VPD, without the *N. antarctica *dominated successional stages, is described by a GAM (VPD [hPa] ~ s (elevation [m], *k* = 5); adj. *R*^2^ = 0.40, *n* = 28), *p* < 0.004) and displayed in Fig. 6. A comparison between the fitted regression line and the occurrences of *N. antarctica* vegetation types showed that a considerable proportion has significantly increased VPD values. All these sites are in the transition zone between *N. dombeyi* and *N. pumilio* vegetation types. Furthermore, the strong effect of the aspect and the continentality gradient (see Fig. 3) became obvious, with only north-facing slopes predominant in the eastern sector (2–4) exceeding the 95% confidence intervals and only south-facing slopes far below the modelled VPD. The forest fire ignition threshold (supplement Figure SF6) according to Paritsis et al. (2015) showed a comparable pattern. We found the highest number of days exceeding the threshold for *A. chilensis* (7.8 ± 2.8), followed by *N. antarctica* (3.3 ± 1.8) and *N. dombeyi* (2.5 ± 2.1), and almost no days with increased fire risk (0.5 ± 1.1) for *N. pumilio* forest stands (mean ± standard deviation). Although *A. chilensis* and *N. dombeyi* occur at the same elevation, the VPDs indicate the significantly higher vulnerability of *A. chilensis* stands to forest fires (Fig. 6).

## Discussion

### Zonation along the gradients

The recorded temperatures of the micro-loggers show a strong linear dependency on elevation. We also observed only minor differences of the elevation-dependent lapse rates between the slope aspects, which is in line with previous observations on slope aspect comparisons (Körner and Paulsen 2004). Therefore, we propose an elevation-dependent temperature lapse rate of 0.47 K 100 m^−1^ for the studied region. This lapse rate is at the lower level but within the range observed globally for temperate mountain forests of appr. 0.5 K 100 m^−1^ (Moser et al. 2009; Barry 2010; Chen et al. 2018). However, our measurements are considerably lower than 0.86–1.06 K 100 m^−1^ reported by Hertel et al. (2008) for *N. pumilio* stands in southern Patagonia. In general, steep lapse rates are caused by dry air, cool temperatures, and high solar radiation inputs (Barry 2010). Further, it is well known that temperature lapse rates are subject to daily and seasonal variations (e.g. Blandford et al. (2008), Tercek et al. (2021)). In contrast, information on inter-annual changes of lapse rates is rare (Kirchner et al. 2013) and could be potentially connected to large-scale climate patterns such as SAM and ENSO. However, the distinct differences in lapse rates within a species distribution range, as shown for *N. pumilio,* and the temporal variability highlight the importance of local-derived bioclimatic indicators of our study.

The characterisation of the longitudinal gradient, from west to east, in terms of continentality (Fig. 3) showed that the longitudinal valley (Fig. 1, 71° 30′ W) represents a significant cut in the proposed gradient. With the division and selection of the four sectors, we have a strong gradient from the most westerly (sector-1) to the second sector, and then rather uniform sampling sites with a slow transition to the semi-arid steppe of northern Patagonian in the east. This refutes our hypothesis (i) of a linear progression of the thermal continentality and also other climatic conditions along the longitudinal gradient.

From the climatic zonation in Fig. 2, we can derive a description of the thermal niche of the species studied, which are hardly given (Amigo and Rodríguez Guitián 2011). One of the rare cases is given by Schlatter (1994) for *N. pumilio* in Chile, with a temperature range for the upper distribution limit of 3.5 to 5 °C and for the lower limit of 6.5 to 7 °C depending on the latitude. These values fit very well with our measurements. Additionally, Weinberger (1973) reported thermic niches for several tree species in Chile, covering also the latitudes of our study area. These climatic niches are described by MDT and mean DRT at 25 cm above forest ground for the warmest quarter of the year (WQ, December to February). Except for *N. antarctica*, all our measurements (Table 1) are at the warmer edge or higher than Weinberger’s values but are not fully comparable due to methodological differences. Thus, it is particularly important to identify the separating thermal conditions for species with overlapping MAT-values, namely *A. chilensis* and *N. dombeyi*. We propose that these two species differ greatly by the mean daily temperature (WQMDT) and the diurnal range of temperature (WQDRT) of the warmest quarter of the year (Table 1, supplement Figure SF2). This is a combination of the self-designed microclimate of the forest stand due to species traits (e.g. leave area index), differences in stand density and structure and the tolerable upper temperature range of the species. The significantly lower DRT values of *N. dombeyi* in comparison to *A. chilensis* (Table 1) indicate a clearly balanced forest interior climate for the former. In a nutshell, the ecosystem engineering efforts of the evergreen mesomorphic *N. dombeyi* are directed towards pronounced oceanic microclimates providing habitat for moist-adapted species of Valdivian origin, whereas *A. chilensis* stands resemble xerophilous, sparse continental Mediterranean coniferous forests already providing habitat for steppe species (Amigo and Rodríguez Guitián 2011). In summary, these results confirm our hypothesis (iii) of distinct niches for vegetation types and provide information on thresholds for species distribution models.

### Disturbance caused micro- and mesoclimatic variations

In our considerations of the microclimatic variations, we focus on two spatial extents and associated disturbance regimes. On the one hand, small-scale gaps are caused by treefall, and, on the other hand, large-scale patches with successional stages dominated by *N. antarctica* are caused by forest fires.

Thereby, the observed thermal niche of *N. antarctica* (Fig. 2) between *N. dombeyi* and *N. pumilio* is in line with the findings of Kalela (1941) and Kitzberger et al. (2022) who stated that the most susceptible sites for forest fires are at the transition zone between the forests dominated by these two species. Paritsis et al. (2015) recorded up to 4 K higher temperatures and an exceedance of the ignition thresholds at post-fire successional stages compared to unburned natural *N. pumilio* forests south of our study region. Even though these great differences could not be confirmed by our study, the signal of higher temperature and altered microclimatic conditions favouring forest fires in *N. antarctica* successional stages are comparable. In conclusion, this indicates that pre-fire forest at the current *N. antarctica* stands had lower temperatures than we currently recorded, and thus, a considerable share of these sites can be attributed to the niche of *N. pumilio*. Furthermore, the successional stages of *N. antarctica* have by far the highest DRT values (Table 1), indicating a strong change of forest interior climate. This in turn has a general unfavourable effect on the regeneration of other *Nothofagus* species and is likely to favour *N. dombeyi*, which has some characteristics of a pioneer tree species in early life stages, over *N. pumilio* (Kalela 1941; Veblen et al. 1996). These findings enable us to prove our hypothesis (iii) regarding inferences on pre-disturbance vegetation types and potential regeneration pathways.

The positive fire feedbacks and consequent shifts from *N. pumilio* and also from *N. dombeyi* forests to fire-prone shrublands are described by Paritsis et al. (2015) and Tiribelli et al. (2018) for Patagonia. To further evaluate this effect, we used the VPD calculated from the recordings of temperature and relative humidity in the forest stands (Fig. 6) and compared it with the VPD values of the *N. antarctica* successional stages. We interpret the observed increased values of the successional stages in mid-elevations as such positive fire feedback and the development of a ‘fire trap’, especially at slopes with northern aspect. Accordingly, shrubland communities are predominantly composed of resprouting species, and its post-fire recovery of biomass occurs swiftly, maintaining a consistently high fine fuel density. This clearly reinforces the evidence of a positive feedback with fire disturbance.

The temporal patterns of the VPD show a bimodal distribution with peaks in midsummer (end of January) and early spring (November) and are especially high for *A. chilensis* and for *N. antarctica* (supplement Figure SF3). During that time of the year, the daily mean values (VPD up to 2.6 kPa) are well above the thresholds given by Clarke et al. (2022) for increased forest fire probability (VPD_50_ 1.3 kPa for temperate broadleaf and mixed forests and 2.3 kPa for Mediterranean forests). This is very well in line with the high forest fire activity in the study region (CAMS 2022). We also found strong similarities between the ignition thresholds of Paritsis et al. (2015) (supplement Figure SF6) and the patterns of VPD (Fig. 6). Our VPD values and interpretation are also in good agreement with the results of Barberá et al. (2023) in a nearby area, although our values are at a lower level.

Regarding the microclimatic variations of the small-scale gaps caused by treefall, the differences were less pronounced than for the comparison of different vegetation types and successional stages. The main reason for that could be the relatively small gap size, as gaps with a diameter up to one tree height can maintain forest conditions (Horváth et al. 2023), and differences in forest structure (De Frenne et al. 2021). Nevertheless, we found significant temperature differences between forest stand and gap for *A. chilensis* in the warmest month of the year (Fig. 5), with up to 2.5 K higher values in the second half of the day (supplement Table ST2). Also *N. pumilio* showed higher temperatures in gaps in the afternoon, although not statistically significant. On warm days, the forests’ interior remains cooler than gaps, which leads to greater differences in DRT (supplement Figure SF5, Table ST3) by means of two mechanisms. Primarily, most of the incoming radiation is absorbed or reflected by the canopy. In addition, the air is cooled as a result of evapotranspiration. Thus, an overheating during the second half of the day is limited (De Frenne et al. 2019). When comparing microclimatic patterns of different seasons, the generally clearer sky conditions during the austral summer (January) and the more overcast conditions during austral winter (July) and its influence on radiation must be taken into account (De Frenne et al. 2021). This can explain the divergent patterns during the coldest months, with only minor differences between gaps and stands (supplement Table ST2, Figure SF5). During the coldest month, the evergreen conifer species, *A. chilensis*, had the highest temperatures in the stand throughout the day, which can be attributed to the lower albedo (increased absorption) and reduced outgoing longwave radiation (increased retention) of the forest stand compared to the gap. For the deciduous broad-leaved species, *N. pumilio*, the patterns were comparable to the one in the summer, suggesting in large parts comparable underlying mechanisms (De Frenne et al. 2021). These results indicate that small-scale gaps have a weaker influence in microclimatic variations than larger scale disturbances, giving partial support to our hypothesis (iii) of altered mesoclimatic patterns after disturbance.

## Conclusion

This study presents fundamental information on the forest interior climatic and microclimatic variations for a distinct part of one of the largest natural deciduous forest areas in the world. We contribute in closing the knowledge gap in terms of climatic information in general and the description of the species climatic niches for mountain forests in Northern Patagonia. Nevertheless, long-term climatological and microclimate monitoring is necessary to capture the interannual variations in the region. Therefore, this study can only be the beginning of the collection of reliable data for this globally remarkable and instructive mountain region.

Our results unveil the characteristic elevational gradient of the species thermal niche characterised by variations in temperature. The presented elevation-dependent lapse rate of the mean annual temperature holds the potential to downscale available coarse-scale climatic information or regionalise local station data. The description of the longitudinal gradient, from west to east, in terms of continentality helped to better characterise the study area for future research questions.

In addition to these pronounced two-dimensional spatial gradients and the associated vegetation zonation, we investigated how disturbance caused microclimatic variations and altered the mesoclimatic pattern. Temperature and diurnal range of temperature differences between forest stands and gaps were more pronounced in the warmest months of the year and at lower elevations, with up to 2.5 K higher temperatures in the second half of the day in gaps. We found clear indications that the successional stage of *Nothofagus antarctica* alters the mesoclimatic pattern, favouring the conditions for forest fire ignition through changes in vapour pressure deficit. Such climatic variations on both spatial levels of disturbance have a major influence on tree species turnover and ecological processes within these forest ecosystems. Thus, this serves as a focal point for future applied research work on mitigating the negative effects of disturbances on the forest ecosystem and its provided services. Tree species selection, tree species proportions and forest structures can be influenced by informed forest management. To support this, we provided different insights into vegetation-climate interactions, possible levers for forest fire risk management and options for mitigating climate extremes by considering the forest interior climate.

### Supplementary information

ESM 1 Supplementary information is available at:https://github.com/simonalois/microclimate_ARG (DOCX 625 kb)
