# Supplementary material for: The interior climate and its microclimatic variation of temperate forests in Northern Patagonia, Argentina
Source: Int J Biometeorol. 2024 Jan 27;68(4):719–30. doi: 10.1007/s00484-024-02617-5 (PMC10963443; doi:10.1007/s00484-024-02617-5)
Supplement: Supplementary file 1 — Supplementary information is available at:https://github.com/simonalois/microclimate_ARG (DOCX 625 kb) [file 484_2024_2617_MOESM1_ESM.docx]

**Supplements**

**The interior climate and its microclimatic variation of temperate forests in northern Patagonia, Argentina**

International Journal of Biometeorology

Alois SIMON^1^, Jonas FIERKE^1,2^, Ernesto J. REITER^3^, Gabriel A. LOGUERCIO^4,5^, Steffi HEINRICHS^1,6^, Birgitta PUTZENLECHNER^2^, Natalia Z. JOELSON^1,7^, Helge WALENTOWSKI^1*^

1 Faculty of Resource Management, HAWK University of Applied Sciences and Arts, Göttingen, Germany

2 Department of Cartography, GIS and Remote Sensing, University of Göttingen, Institute of Geography, Germany

3 Plant Ecology and Ecosystems Research, University of Göttingen, Germany

4 Andean Patagonian Forest Research and Extension Center (CIEFAP), Argentina

5 Faculty of Engineering, Department of Forestry, National University of Patagonia San Juan Bosco, Argentina

6 Silviculture and Forest Ecology of the Temperate Zones, University of Göttingen, Germany

7 Faculty of Biology and Psychology, University of Göttingen, Germany

* corresponding author, email: helge.walentowski@hawk.de

Supplementary information is available at: <https://github.com/simonalois/microclimate_ARG>

Table ST1: Results of the ordinary least square linear regression for the elevation-dependent Mean Annual Temperature (MAT) lapse rate of Figure 2.

| Variable | β_i_ | Standard Error | Significance (two-sided p-value) |
| --- | --- | --- | --- |
| Intercept | 11.994 | 0.3054 | <0.0001 |
| Elevation [m] | -0.0047 | 0.0002 | <0.0001 |

F-Statistic (df=35) 312.9, p<0.0001; Multiple R² = 0.899, adj. R² = 0.896

Table ST2: Summary and statistical comparison of the mean temperatures [°C] between forest stand and gap measurement locations. mean ± standard deviation; p: p-value (two-sided t-test); italic and colour: warmer temperture of the comparison.

|  | *Austrocedrus chilensis* | | | *Nothofagus pumilio* | | | *N. pumilio - N. antarctica* (patch) | | |
| --- | --- | --- | --- | --- | --- | --- | --- | --- | --- |
|  | stand | gap | p | stand | gap | p | stand | patch | p |
| Time | warmest month (January) | | | warmest month (January) | | | warmest month (January) | | |
| 02:00 | *14.8 ± 3.7* | 13.9 ± 3.5 | 0.363 | *9.1 ± 3.9* | 8.2 ± 3.5 | 0.367 | *11.4 ± 4.1* | *10.7 ± 4.1* | *0.492* |
| 06:00 | *12.2 ± 3.3* | 11.2 ± 3.3 | 0.271 | *7.8 ± 3.7* | 6.5 ± 3.3 | 0.149 | *10.1 ± 4.0* | *9.1 ± 3.9* | *0.353* |
| 10:00 | 15.0 ± 3.2 | *17.2 ± 3.5* | **0.014** | *11.1 ± 4.9* | 10.8 ± 4.7 | 0.773 | *12.8 ± 3.9* | *12.5 ± 3.8* | *0.747* |
| 14:00 | 24.3 ± 5.1 | *27.0 ± 5.3* | **0.049** | 17.0 ± 6.0 | *19.0 ± 6.8* | 0.221 | *17.9 ± 4.5* | *19.5 ± 4.9* | *0.191* |
| 18:00 | 24.1± 6.1 | *25.7 ± 6.3* | 0.310 | 17.3 ± 6.3 | *17.7 ± 6.7* | 0.791 | *19.1 ± 5.5* | *20.1 ± 6.1* | *0.494* |
| 22:00 | *18.6 ± 4.5* | 18.2 ± 4.5 | 0.746 | 11.9 ± 4.5 | 11.7 ± 4.6 | 0.877 | *14.0 ± 4.4* | *13.8 ± 4.6* | *0.806* |
| Time | coldest month (July) | | | coldest month (July) | | | *coldest month (July)* | | |
| 02:00 | *1.1 ± 1.4* | 0.8 ± 1.4 | 0.422 | *-2.3 ± 2.3* | -2.8 ± 2.7 | 0.486 | *-0.8 ± 1.9* | *-0.8 ± 2.0* | *0.930* |
| 06:00 | *0.7 ± 1.5* | 0.5 ± 1.5 | 0.474 | *-2.3 ± 2.2* | -2.7 ± 2.3 | 0.486 | *-0.9 ± 1.7* | *-0.8 ± 1.8* | *0.891* |
| 10:00 | *0.9 ± 1.4* | 0.6 ± 1.5 | 0.572 | *-1.8 ± 2.0* | -2.0 ± 2.0 | 0.705 | *-0.6 ± 1.6* | *-0.6 ± 1.6* | *0.921* |
| 14:00 | *4.7 ± 2.8* | 4.1 ± 2.5 | 0.657 | 1.2 ± 2.4 | *2.1 ± 3.4* | *0.227* | *0.8 ± 1.4* | *1.2 ± 1.3* | *0.258* |
| 18:00 | *3.6 ± 2.3* | 3.2 ± 2.1 | 0.545 | -0.6 ± 2.3 | *-0.5 ± 2.7* | *0.924* | *0.2 ± 1.7* | *0.5 ± 1.7* | *0.502* |
| 22:00 | *1.8 ± 1.5* | 1.5 ± 1.4 | 0.315 | *-1.9 ± 2.0* | -2.2 ± 2.3 | 0.563 | *-0.5 ± 1.8* | *-0.4 ± 1.8* | *0.773* |

Table ST3: Summary and statistical comparison of the diurnal range of temperature (DRT) [K] between forest stand and gap measurement locations. mean ± standard deviation; p: p-value (two-sided t-test)

|  | Warmest Quarter DRT | | | Coldest Quarter DRT | | |
| --- | --- | --- | --- | --- | --- | --- |
| species | stand | gap | p | stand | gap | p |
| *A. chilensis* | 12.7 ± 4.8 | 15.5 ± 5.1 | **<0.001** | 5.4 ± 3.2 | 5.4 ± 3.3 | 0.98 |
| *N. dombeyi* | 9.9 ± 4.0 | 12.3 ± 5.3 | **0.002** | 3.9± 2.5 | 5.3 ± 3.9 | **0.023** |


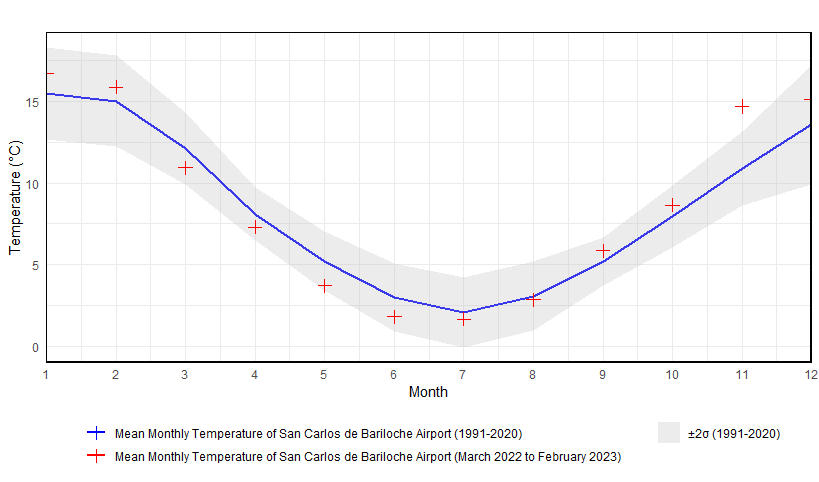


Figrue SF1: Mean Annual Temperatur (MAT) of the meteorological station at the San Carlos de Bariloiche Airport (842 m a.s.l.; latitude: -41.151, longitude: -71.158) over the last 30-year climate reference period (1991-2020) with the MAT of the recording period.


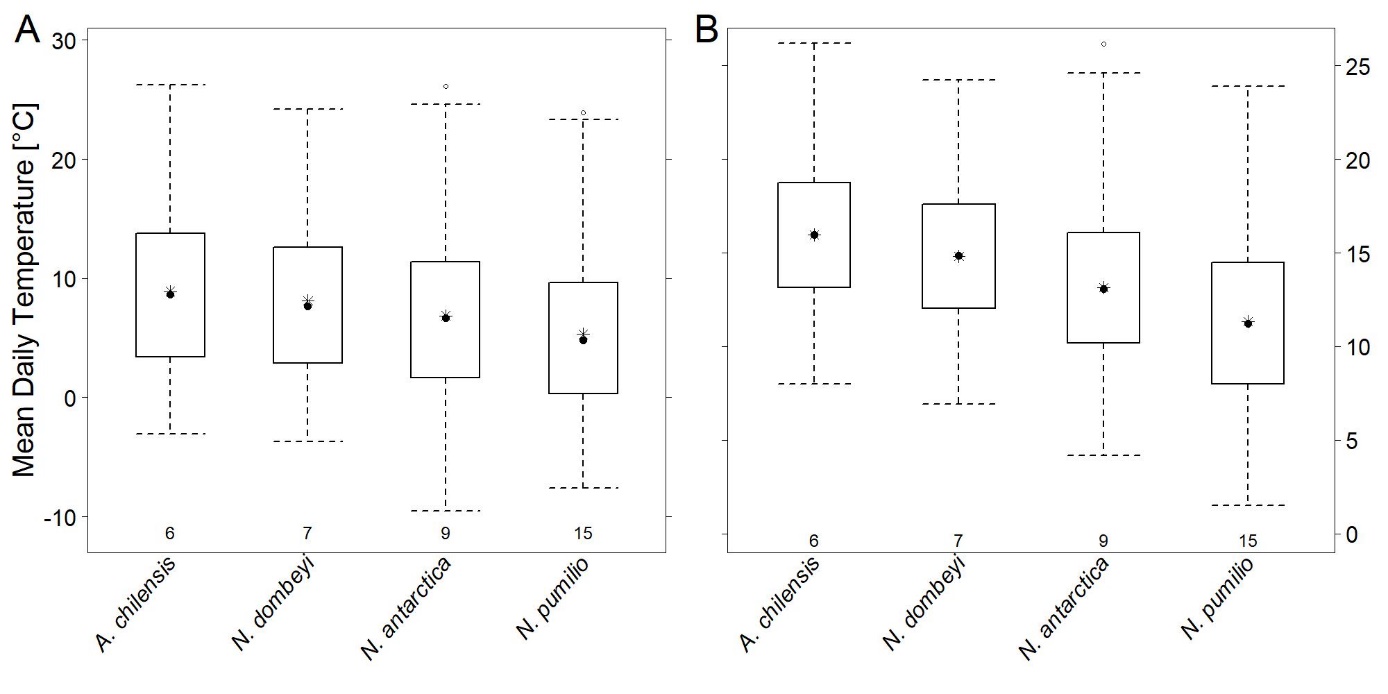


Figure SF2: Forest interior temperature differentiation between main vegetation types for the whole measurement period (A) and for the warmest quarter (WQMDT) (B). points: median, asterisk: mean; whisker: maximum of 1.5xinterquartile range, numbers indicate count of recordings.


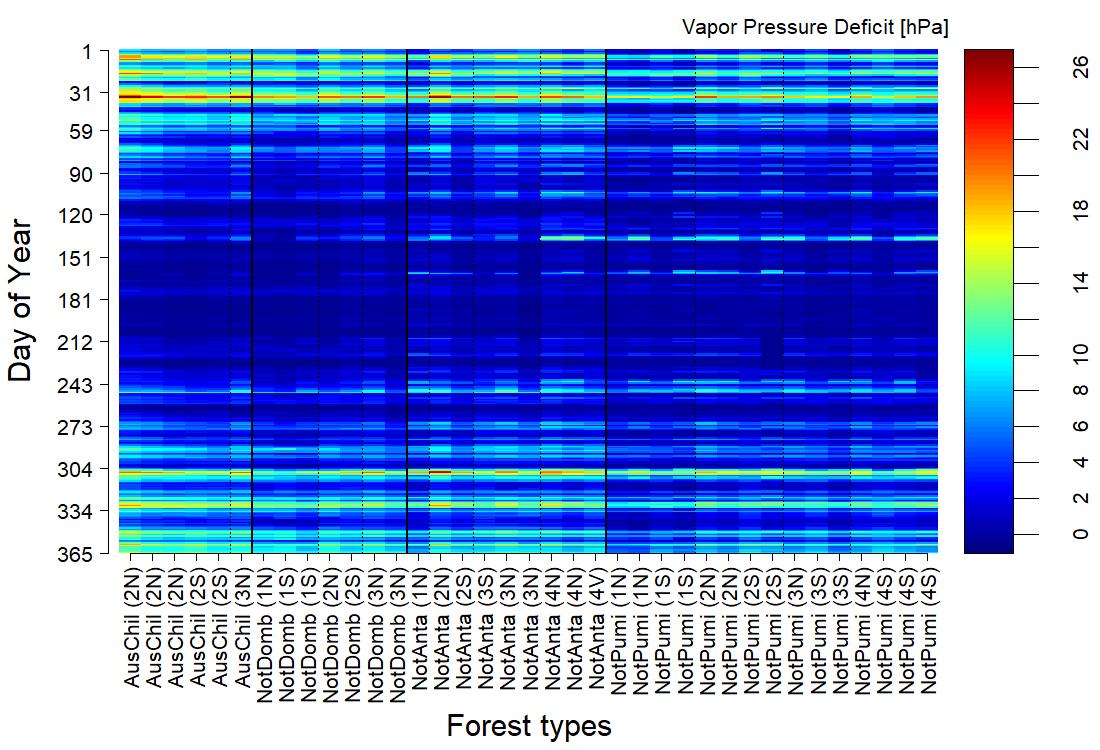


Figure SF3: Temporal pattern of the daily mean Vapour Pressure Deficit (VPD) of the different vegetation types (without gaps). Yaxis: AusChil: *A. chilensis,* NotDomb: *N. dombeyi,* NotAnta: *N. antarctica*, NotPumi: *N. pumilio*; in brackets: sector (1-4) and aspect (N: north, S: south, V: valley bottom); sorted by sector (dashed line) and elevation; see also Figure 1


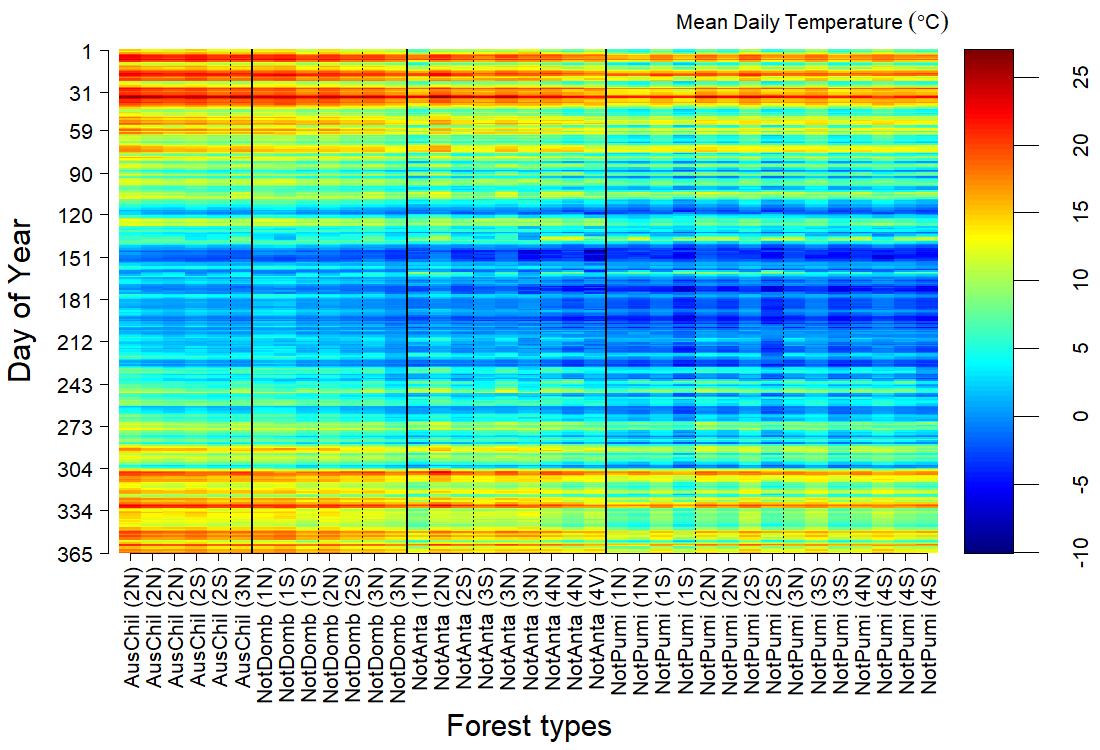


Figure SF4: Temporal pattern of the Mean Daily Temperature (MDT) of the different vegetation types (without gaps). Xaxis: AusChil: *A. chilensis,* NotAnta: *N. antarctica*, NotDomb: *N. dombeyi,* NotPumi: *N. pumilio*; in brackets: sector (1-4) and aspect (N: north, S: south, V: valley bottom); sorted by sector (dashed line), aspect and elevation; see also Figure 1


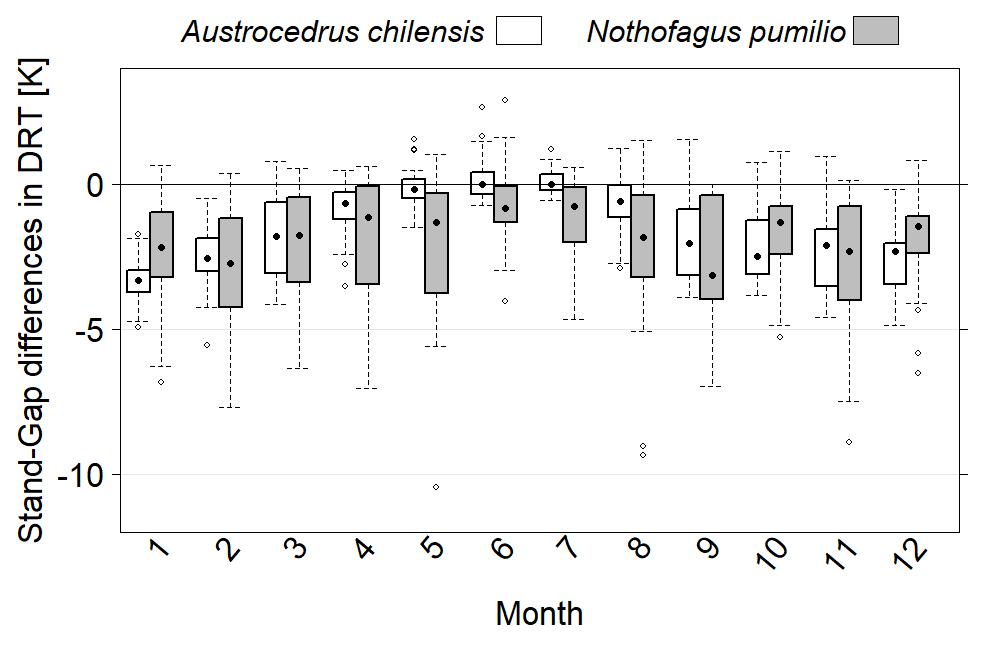


Figure SF5: Annual pattern of the differences in Diurnal Range of Temperature (DRT) between forest interior climate and gaps. full points: median; whisker: maximum of 1.5xinterquartile range


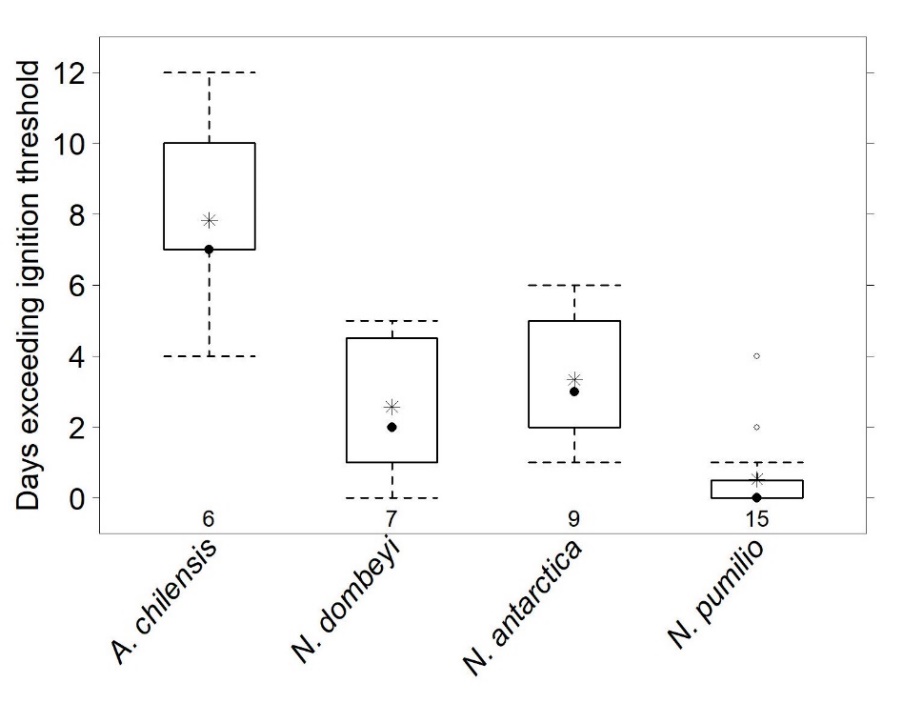


Figure SF6: Number of days with maximum daily temperature >25°C and minimum relative humidity <25% as threshold for increased forest fire ignition according to Sagarzazu and Defossé (2009). points: median, asterisk: mean; whisker: maximum of 1.5xinterquartile range, numbers indicate count of recordings.
